# Cost-effectiveness of group-based exercise to prevent falls in elderly community-dwelling people

**DOI:** 10.1186/s12877-021-02329-0

**Published:** 2021-07-26

**Authors:** Benjamin Scheckel, Stephanie Stock, Dirk Müller

**Affiliations:** Institute of Health Economics and Clinical Epidemiology, Faculty of Medicine and University Hospital Cologne, University of Cologne, Gleueler Str. 176-178, 50935 Cologne, Germany

**Keywords:** Cost-effectiveness, Markov model, Hip fracture, Fall prevention, Elderly people

## Abstract

**Background:**

Clinical studies indicate that strength-balance training for active fall prevention can prevent fractures in older people. The present modelling study evaluates the cost-effectiveness of fall prevention exercise (FPE) provided to independently living older people compared to no intervention in Germany.

**Method:**

We designed a Markov model to evaluate the cost-effectiveness of a group-based FPE-program provided to independently living people ≥75 years from the perspective of the German statutory health insurance (SHI). Input data was obtained from public databases, clinical trials and official statistics. The incremental cost-effectiveness ratio (ICER) was presented as costs per avoided hip fracture. Additionally, we performed deterministic and probabilistic sensitivity analyses and, estimated monetary consequences for the SHI in a budget impact analysis (BIA).

**Results:**

For women, the costs per hip fracture avoided amounted to €52,864 (men: €169,805). Results of deterministic and probabilistic sensitivity analyses confirmed the robustness of the results. According to the BIA, for the reimbursement of FPE additional costs of €3.0 million (women) and €7.8 million (men) are expected for the SHI.

**Conclusions:**

Group-based FPE appears to be no cost-effective option to prevent fall-related hip fractures in independently living elderly. To allow a more comprehensive statement on the cost effectiveness of FPE fracture types other than hip should be increasingly evaluated in clinical trials.

**Supplementary Information:**

The online version contains supplementary material available at 10.1186/s12877-021-02329-0.

## Introduction

An increased risk of fall in higher age (i.e., > 75), is associated with both physical and mental restrictions for the affected individuals and, may result in premature death [1–3]. Falls can result in fractures at different sites, with proximal femur, pelvis, distal radius ankle and proximal humerus diagnosed most often in individuals aged between 70 and 89 years [4]. Fall-related fractures have significant socioeconomic consequences for both patients and the society. Particularly, hip fractures can lead to a substantial loss of healthy life-years in elderly people [5]. In the European Union, the costs of fall-related hip fractures, which are more than 90% of all hip fractures [6] amounted to €20 billion in 2010 [7, 8]. Particularly, elderly people with low bone density are at increased risk of hip fracture. Due to demographic changes, in the European Union, the number of osteoporosis-associated hip fractures is expected to increase from 615,000 in 2010 to 815,000 in 2050 (+ 32%) [8].

Among various measures to prevent falls and fall-related fractures, exercise is based on the knowledge that physical inactivity in older age increases the likelihood of suffering a fall-related fracture [9]. For the mode of action of exercise different mechanisms are discussed: exercise can prevent fractures from falls by improving balance and muscle strength, but also by strengthening muscles and/or increasing bone mineral density [10]. Therefore, for fall prevention exercises (FPE) it is recommended to include different modules of exercises such as improving balance with the addition of strength training and/or walking [11]. In addition, FPE should be carried out on a regular basis [11]. Structured, commonly used FPE programs are the Falls Management Exercise Program (FaME) and the Otago Exercise Program (OEP) [12].

Whereas high-level evidence for preventive measures is often difficult to achieve, FPE provided to elderly in community-based settings has been shown to be effective in reducing falls and fall-related fractures [11, 13, 14]. In addition, by providing group-based FPE many individuals can be targeted simultaneously.

Different cost-effectiveness analyses of exercise for fall prevention in different settings have been published, ranging from economic evaluations alongside clinical trials to modelling studies [15–19]. However, only few studies evaluated group-based FPE as a single intervention for community-dwelling elderly with regard to long-term consequences [15, 19, 20].

The objective of this analysis was to evaluate the cost-effectiveness (i.e., the costs per hip fracture avoided) of group-based FPE for elderly persons without care need compared to no intervention from the perspective of the German statutory health insurance (SHI). Furthermore, we estimated the monetary consequences of reimbursing such a program for the SHI.

## Methods

We performed a cost-effectiveness analysis with a Markov-based simulation using TreeAge Pro© (TreeAge Software, Williamstown, Massachusetts). The model cohort, i.e. community-dwelling older people at average risk of hip fracture, entered the model at the age of 75 and was tracked for 25 years up to the age of 100 years. This age was chosen due to a lack of public data on German women and men older than 100 years. The chosen cycle length was 6 months because hip fractures are often followed by a re-fracture, nursing home admission or death within the first months post-fracture [21, 22].

For input data on clinical parameters and costs, different literature searches were performed in bibliographic databases (e.g., Medline) and public sources (e.g., Federal Statistical Office of Germany). Finally, most of the data for populating the model were based on German sources, supplemented by studies from other European countries. The appropriateness of the data applied for the model was checked by clinical experts. The primary outcome of this analysis was the incremental costs per avoided hip fracture. The cost-effectiveness-analysis was limited to hip fractures because these are expected to have the largest socioeconomic impact. In addition, fractures other than hip were often not reported in clinical trials [23].

### Overview of the model structure

**Fig. 1:**
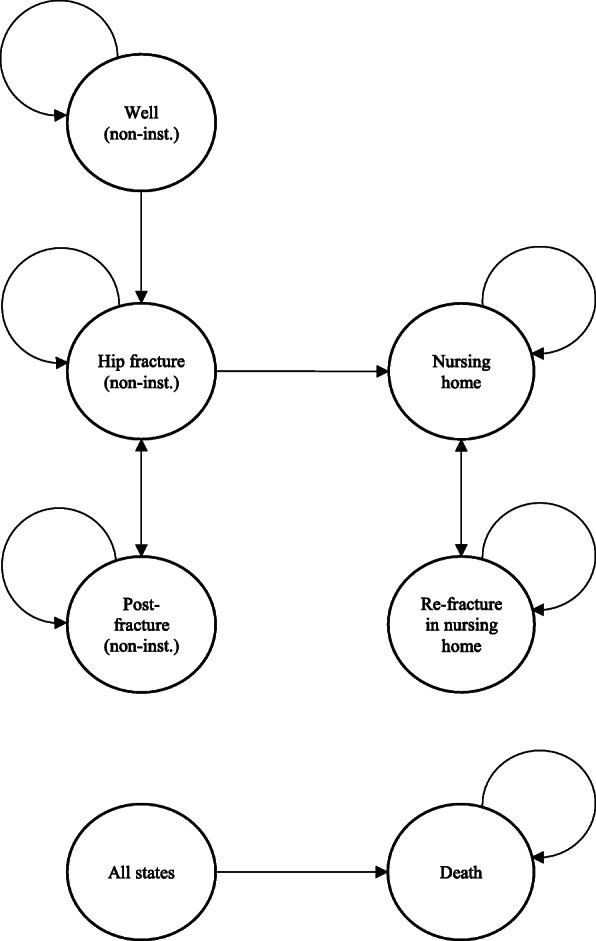
Transition state Markov model for the base-case analysis [24]. Abbreviations. Non-inst.: non-institutionalized

Our analysis was based on a previous Markov-model developed for a cost-utility-analysis of a home-safety intervention for older people in need of care [24]. The model included six health states: “well”, “hip fracture”, “post-fracture”, “nursing home”, “re-fracture in nursing home” and “death”. Women/men entered the model in the state “prior first hip fracture”, could remain in this state, suffer a hip fracture or die. (Note: deaths related to a hip fracture or occurring in the nursing home are considered separately in the further progress of the model.) If a first hip fracture occurs, subjects can return to home (state “post-fracture”), be admitted to nursing home (state “nursing home”), suffer a re-fracture or die. In case of a re-fracture, patients remain in the state “hip fracture”. Transitions to nursing home due to hip fracture were assumed only to occur in the first cycle after a hip fracture (compare Fig. 1). When admitted to nursing home, patients could stay in nursing home, suffer a re-fracture of the hip, or die.

### Intervention

Among the several concepts for offering an FPE-program, two types are recommended: individualized home-based training and group-based exercises, both of them including strength- and balance-training [11]. Because of the group-based nature of most commercial FPEs in Germany, for our model a standardized group exercise was applied. We assumed FPE to be provided on a weekly basis, corresponding to the intervention frequency from an offer of a German hospital [25]. In accordance with similar studies, the program was assumed to include 24 one-hour sessions [12, 26]. In these sessions, the participants were assumed to receive different exercises and, specific guidance how to practice the exercises at home.

### Clinical input data

Data on hip fracture rates were derived from a large claim data set from Southern Germany. In that analysis, incidences of femoral fractures (ICD-Code S72) in different care settings were analysed [27]. Hip fracture rates for the model population were calculated by limiting the number of femoral fractures to hip fractures, which were assumed to be 84% of all femoral fractures (i.e. ICD-Code S72.0-S72.2), and, by subtracting the proportion of fractures not resulting from a fall [28–30]. Risks of a re-fracture were taken from a large Danish population-based cohort study [22]. This study revealed a substantial increased relative risk of a re-fracture, which realigned within 20 years of follow-up. We applied these re-fracture risks also to individuals admitted to nursing home because of a lack of evidence for this subgroup.

Rates of nursing home admission after hip fracture were taken from insurance data reflecting community-dwelling people with a hospital admission or discharge diagnosis of a hip fracture (S72.0-S72.2) [21].

Data on mortality in the “prior first hip fracture”-state were taken from the Federal Statistical Office of Germany [31]. Both the increased short-term mortality in the first cycle after a hip fracture (state “hip fracture”) [21] and the increased long-term mortality after returning home (state “post-fracture”) was taken into account [32]. Data on short-term and long-term mortality after nursing home admission were taken from claims reflecting German nursing home residents [33] (Table 1).

**Table 1:** Clinical input data (base-case analysis)

Age (years)	Hip fracture (%, non-inst., w/m) [27, 30, 34]	Re-fracture (%, non-inst., w/m) [22, 27, 30, 34]	Re-fracture (%, nursing home, w/m) [22, 30, 34, 35]	Nursing home admission (%, w/m) [21, 30]	Pre-fracture Mortality (%, non-inst., w/m) [28, 30, 31, 33, 36]	Hip-fracture mortality (%, non-inst., w/m) [21, 30, 32]	Mortality (%, nursing home, w/m) [33, 35, 37]	Re-fracture mortality (%, nursing home, w/m) [33, 35, 37]
		Months 1–6^a^ (Months 7 + ^a^)	Months 1–6^a^ (Months 7 + ^a^)	Months 1–6^a^		Months 1–6^a^ (Months 7 + ^a^)		Months 1–6^a^
**75–79**	0.003/0.002	0.018/0.009	0.064/0.030	0.067/0.066	0.009/0.018	0.038/0.082	0.134/0.224	0.252/0.435
		(0.004/0.002)	(0.019/0.013)			(0.018/0.032)		
**80–84**	0.006/0.003	0.034/0.017	0.079/0.043	0.117/0.096	0.016/0.030	0.056/0.109	0.134/0.223	0.251/0.433
		(0.009/0.004)	(0.023/0.018)			(0.023/0.045)		
**85–89**	0.010/0.005	0.055/0.027	0.084/0.039	0.147/0.106	0.029/0.047	0.073/0.13	0.161/0.266	0.298/0.501
		(0.014/0.007)	(0.027/0.021)			(0.042/0.069)		
**90–94**	0.015/0.009	0.075/0.043	0.091/0.047	0.180/0.117	0.063/0.074	0.129/0.188	0.161/0.265	0.297/0.500
		(0.020/0.012)	(0.029/0.025)			(0.076/0.101)		
**95+**	0.019/0.012	0.094/0.058	0.069/0.015	0.201/0.186	0.063/0.074^b^	0.129/0.188^b^	0.217/0.332	0.389/0.597
		(0.025/0.016)	(0.025/0.023)			(0.076/0.101^b^)		

Confidence intervals (CI) were not reported for all clinical parameters in the literature. Where no 95%-CI was available, we assumed a range of +/− 20%

*Abbreviations*. *w* Woman, *m* Men, *not-inst*. Non-institutionalized

^a^Post hip fracture

^b^There were only data for 90+ available, so these probabilities are identical with the ones for 90–94

A recent Cochrane review showed FPE to be effective in elderly people living without care in the community for the prevention of fractures [23]. The risk ratio (RR) of suffering at least one fracture was 0.73 [95% Confidence interval (CI): 0.56 to 0.95] compared to standard care or no intervention [23]. For our analysis, we adjusted this risk reduction for inadequate compliance (i.e., conforming to the recommendations) and non-persistence (i.e., conforming to a recommendation of continuing treatment for the prescribed length of time) [38]. Because some individuals may reject the offer of group exercise the intervention effect was reduced in accordance with the number of participants not taking part in the intervention [23, 28].

In order to take into account non-persistence, we calculated a proportional effect decrease of 28% annually [23, 39]. As a result, after 4 years the effect of the FPE was assumed to be zero.

### Cost data

In accordance to the SHI-perspective, we included the costs of FPE, those of hip-fracture-related treatment and nursing. The costs assumed for the provision of FPE were in line with the requirements for fall prophylaxis training as defined by SHI, i.e., participants pay registration fees in advance and will receive up to 80% of the costs back by the SHI in case of regular participation [25, 40, 41]. Similar to the decreasing effect of the intervention, a yearly reduction of costs due to decreasing compliance and persistence was assumed (Table 2).

**Table 2:** Cost data (base-case analysis)

	Age (years)	Value^a^	Reference
**Costs of intervention**^b^		w/m, €/year	
	75	139/139	[25, 28, 39, 40]
	76	93/94	
	77	49/49	
	78	5/5	
	79+	0/0	
**Costs of hip fracture treatment**^c^		€/fracture	
Hospital care	All ages	7280	[42–44]
Revision	All ages	961	[42–45]
Rehabilitation	All ages	2209	[28, 44, 46–49]
Outpatient Care	All ages	1114	[44, 50–53]
**Costs of long-term care**^c^		w/m, €/6 months	
Non-inst. (prior hip fracture)	75–79	394/348	[28, 36, 42, 54]
	80–84	831/651	
	85–80	1,60/1.221	
	90+	2550/2000	
Non-inst. (post hip fracture)	75–79	990/918	[28, 36, 42, 54]
	80–84	2264/2000	
	85–80	3274/2748	
	90+	4519/3764	
Nursing home^d^	All ages	8516/8516	[28, 54]

*Abbreviations*. *Non-inst*. Non-institutionalized, *w* Women, *m* Men

^a^Since standard deviations were not available on the literature, we assumed a deviation of 40% for treatment costs and long-term care costs, and 50% for the intervention costs [55]

^b^Intervention costs decreas yearly by 28% due to decreasing adherence and additional by the age-specific care rate

^c^For details on calculation see appendix Table A1 and A2

^d^Long-term care costs in the not-institutionalized setting were calculated by multiplying the age-specific care rate with an average value for long-term care costs (for details on calculation see appendix Table A3-A6)

In order to take into account hip-fracture-related home care needs, the corresponding costs were estimated based on different care level rates [29, 42, 54]. For patients returning to home after a hip fracture, we assumed an increase of average care dependency of 9% in the age-group of 75–79 years and an increase of 22% in older age-groups [36]. Costs due to nursing home admission were calculated based on long-term care insurance statistics and the benefit claims per level of care (Table 2) [28, 54]. A nursing home admission was assumed to be irreversible (i.e., short-term stays were not considered).

Costs of hip fracture treatment include costs of inpatient treatment, outpatient treatment and rehabilitation. Data on surgical procedures for hip fracture treatment were taken from published evidence (S72.0-S72.2) [42]. The corresponding amounts of reimbursement to German hospitals were calculated with a freely available DRG-grouper [43]. Costs for inpatient rehabilitation were estimated on SHI statistics [46–48] while outpatient care costs were recalculated from a German cost-utility analysis [50]. Details on cost categories and calculation can be found in the appendix (Table A1 and A2).

Costs were reported in Euro (€). If costs occurred earlier than 2019, we adjusted for inflation (Table 2) [49].

### Sensitivity analysis

We performed different sensitivity analyses with regard to input data and the model structure. For cost data, ranges of 40% as suggested by Briggs were used (except for the intervention costs, where we assumed a range of 50%) [55]. For transition probabilities without a 95%-CI, we used ranges of +/− 20%. For the effect measure, we used the 95%-CI.

Additionally, best- and worst-case analyses were applied. Best-case analysis included the lowest intervention costs and highest costs of treatment and nursing care post-fracture as well as the lower bound of the 95%-CI of the effect measure. In contrast, in the worst-case analysis we applied the highest intervention cost and, the lowest costs of treatment and nursing care costs post fracture as well as the upper bound of the 95%-CI of the effect measure.

Using appropriate distributions for each parameter, a Monte Carlo simulation with 10,000 iterations was performed to quantify the uncertainty of the input parameters. Distribution parameters were calculated from available 95%-CIs or from assumed standard deviations [55]. Treatment and nursing home costs were calculated independent on age, while costs of outpatient care and intervention costs were calculated for each age group. Results of the Monte Carlo simulation were presented as cost-effectiveness acceptability curves.

With regard to the chosen decision model, two structural sensitivity analyses were performed. In the structural analysis I, we added a transition for admissions to nursing home due to other reasons than hip fractures (i.e., from the “healthy” and the “post-fracture” state) [56]. In the structural analysis II, two health states for reflecting the economic impact of vertebral fractures were added (resulting in a ratio ‘costs per hip or vertebral fracture avoided’) [57, 58]. Illustrations of model structures and details on additional input parameters can be found in the appendix (figure A1 and A2, Table A7 and A8).

### Budget impact analysis

Furthermore, we calculated the budget impact of a nationwide implementation of the training program for the German SHI. In order to calculate this budget impact analysis (BIA), the incremental costs were extrapolated with the number of independently living individuals based on their remaining life expectancy [29, 59]. Results are reported as annual costs for the SHI.

## Results

For women aged 75, the provision of FPE resulted in an ICER of 52,864 € per hip fracture avoided. For men at this age, the ICER was €169,805 per hip fracture avoided (Table 3). FPE decreased the number of nursing home admissions due to hip fractures (0.02% for women and 0.01% for men) (Table 3).

**Table 3:** Results of the base case analysis

	Costs (€)		Hip fractures (re-fractures)^b^	Admissions to nursing home^b^	ICER (€/avoided hip fracture)
**Women**					
FPE	31,829 − Intervention: − Fracture treatment^a^: − Long-term care:	268 2,153 29,408	0.1888 (0.0394)	0.0405	
No FPE	31,682 − Fracture treatment^a^: − Long-term care:	2,193 29,489	0.1916 (0.0401)	0.0407	
Δ	148		0.0028	0.0002	
					52,864
**Men**					
FPE	16,986 − Intervention: − Fracture treatment^a^: − Long-term care:	266 822 15,898	0.0716 (0.0066)	0.0114	
No FPE	16,758 − Fracture treatment^a^: − Long-term care:	842 15,916	0.0729 (0.0067)	0.0115	
Δ	228		0.0013	0.0001	
					169,805

^a^Hip fracture treatment includes hospital treatment, revision, rehabilitation and outpatient care

^b^Values are proportion

*Abbreviations*. *FPE* Fall-prevention exercise, *Δ* Difference between FPE and no FPE, *ICER* Incremental cost-effectiveness ratio

### Sensitivity analysis

For both women and men the efficacy of FPE had the highest impact on the result, followed by the intervention costs. When varying the efficacy of FPE, the costs of the intervention or, the fracture rates, for men the impact on the ICER was substantially higher than for women (see appendix, figure A3 and A4). Assuming a best-case scenario, for women FPE was dominating (i.e., less costly and more effective) compared to no FPE.

**Fig. 2:**
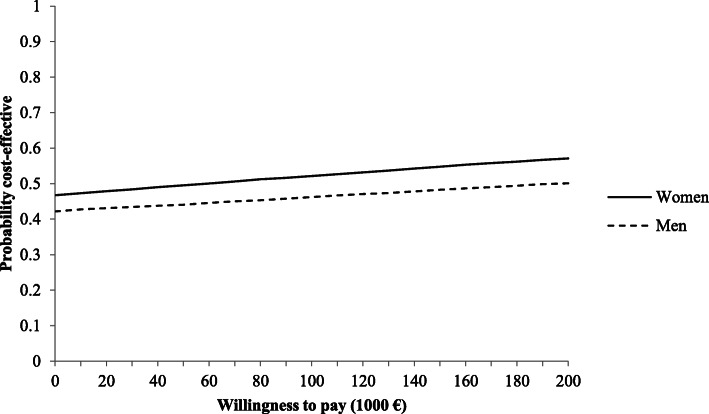
Cost-effectiveness acceptability curve

Assumed a willingness to pay (WTP) of zero, the probabilistic sensitivity analysis showed a probability of 47% for FPE being cost-effective in women. The probability increased to 57% at a WTP of 200,000€. For men, the probability of cost-effectiveness starts at 42% at a WTP of zero and amounts to 50% at a WTP of about €500,000 (Fig. 2).

Incorporation of additional transitions to nursing home unrelated to hip fractures (structural sensitivity analysis I) increased the ICER by €7000 (13%) for women. For the men there was an increase of about €2400 (1%) on the ICER. The inclusion of vertebral fractures in the model (structural sensitivity analysis II) decreased the ICER by 12% (€6200) for women and by 54% (€91,000) for men (see appendix, Table A9).

The budget impact analysis showed that the provision of FPE to independently living community-dwelling adults aged 75 ≤ would be associated with annual costs of €3 Million (Mio) for women and €7.8 Mio for men.

## Discussion

### Summary of the main results

According to the results of this cost-effectiveness analysis group-based FPE for independently living older people cannot be considered as cost-effective. The provision of a FPE program revealed a cost-effectiveness ratio of €52,864 (women) and €169,805 (men), respectively. FPE provided for independently living people ≥75 years would result in additional costs of about €11 Mio for the German SHI. In order to achieve a probability of cost-effectiveness of at least 50%, the WTP had to be €100.000 for women and €200.000 for men. However, a structural sensitivity analysis indicated that the cost-effectiveness ratios for FPE would be more favourable when vertebral fractures (or other type of fractures) would be considered.

The unfavourable cost-effectiveness ratios of group-based FPE can be explained by three reasons: first, the low hip fracture rates in the population of independently living people (seven per 1000 women and four per 1000 men) [27]. Second, a conservative assumption for the data on efficacy of FPE by assuming a continuous decrease due to non-compliance and non-persistence. Finally, the exclusion of non-hip fractures due to a lack of data on efficacy of FPE for these fractures.

### Strengths of our analysis

Overall, the data applied for the model appropriately reflects the care of elderly patients with hip fractures. Particularly, data on hip fractures was obtained from high-quality evidence or from German sources (e.g. [27]), ensuring a sufficient level of representativeness. By focusing on hip fractures, the model highlights the most relevant consequence of a fall from a socioeconomic perspective. Clinical data revealed that hip fractures are the major cause of disability, nursing home admission and mortality [60]. Moreover, for relevant input parameters we could rely on country-specific claims data with large samples [27, 28, 35]. Where no data were available for Germany, we could take data from European countries with a similar standard of care [22, 32]. A further strength of this analysis is in the consideration of transitions to nursing home due to hip fractures (including the increased risk of re-fractures in a nursing home setting).

### Limitations

By using a modelling approach, different structural and parameter-related constraints may limit the validity of the results. First, the exclusion of non-hip fractures due to a lack of high-quality data may result in an underestimation of the cost-effectiveness of FPE. Although we met this limitation by incorporating vertebral fractures in a structural sensitivity analysis – which improved the ICER by 12% (women) and 54% (men) - other clinical events such as pelvic and wrist fractures or even traumatic brain injuries were not reflected in the model. In addition to the prevention of non-hip-fractures and other injuries, further potential health gains of FPE are in benefits to mental health resulting from social contact in a group, reduced depressive symptoms or less fear of falling [61]. Including these aspects is likely to improve the cost-effectiveness of FPE in independently living elderly. Moreover, the occurrence of cardiovascular events such as stroke would be positively affected by active training-sessions such as FPE [62].

Second, in the model, a transition to nursing home is only possible immediately after a hip-fracture. Whereas the impact of nursing home transitions unrelated to hip fractures was evaluated in sensitivity analyses, the number of delayed nursing home transitions after hip fractures is unknown. Consideration of delayed nursing home transitions because of a hip fracture may further improve the cost-effectiveness of FPE.

Third, compared to all nursing residents, for patients with a hip fracture who are admitted to nursing home, an increased mortality was based on claims data for the first 6 months post-fracture [21]. A persistent increase of mortality (i.e. > 6 months post-fracture) was not modelled due to a lack of data. However, variation of mortality parameters as part of the sensitivity analysis in the states post hip fracture resulted only in minor changes of the cost-effectiveness ratio.

Fourth, the costs of FPE were based on payments of the German SHI, which may differ from the real resource consumption. For example, Deverall et al. showed significant cost differences between a home-based, a peer-led group-based and a commercial provided group-based FPE [20]. In addition, the transferability of our results to other health care systems might be limited.

Finally, there is inconclusive evidence of adherence of FPE for the time beyond the study period (> 1 year). We conservatively assumed a 28% reduction of adherence per year which might still underestimate the real proportion of dropouts [39]. However, the assumed efficacy of FPE was based on a wide range of clinical trials in different settings [21].

### Comparison with previous studies

Previous economic modelling studies, which evaluated the cost-effectiveness of FPE, differed in multiple methodological aspects. In addition, the reported results varied markedly:

Assessing balance and strength exercise provided to community living elderly, the analysis of Frick et al. resulted in an ICER of about €115,000 per QALY gained (compared to standard care) [19, 63]. Church et al. compared different strategies for fall prevention [15]. For the comparison of group-based exercise to no intervention in the general population, there was an ICER in the amount of €2964 per avoided fall [15, 63]. However, the probability of group-based exercise being cost-effective was about 10% at a WTP of 100,000. Deverall et al. compared three different types of exercise interventions [20]. As a result, a home-based exercise program was most cost-effective compared to no intervention with an ICER of €3594 per QALY gained, followed by a peer-led group-based variant (€7343) and a commercially provided group-based exercise (€26,665) [20, 63].

To some extent, the differences between the compared models result from the definition of outcome parameter (e.g. injuries, fractures or falls) and, the perspectives used for the analyses. In addition, the analyses targeted specific subgroups without using a well-established and standardized framework for determining a patient’s risk of fall [15, 20].

### Future research

To obtain more reliable information about the cost-effectiveness of FPE, more data on clinical effectiveness are needed. Particularly, clinical trials addressing fractures other than hip would enable researchers to draw more robust conclusions. Furthermore, future trials should be sufficiently powered with fractures as primary outcome. Additionally, the inclusion of health gains beyond fracture prevention (e.g., benefits to mental health or less fear of falling) is desirable to provide more robust data on these outcomes [20, 64].

Because FPE is expected to result in a significant reduction of fractures over years but not months, future clinical studies also should reflect a sufficient period of observation. For all trials addressing fracture prevention, the attenuation of the treatment effect due to e.g. decreasing adherence should be taken into consideration.

## Conclusions

Our analysis suggests that FPE delivered in a group-based setting may have only little impact for the prevention of fall-related hip fractures and the associated costs. Because of a higher fracture incidence in women compared to men, FPE in women has a more favourable cost-effectiveness ratio. For both women and men, our results show an improved cost-effectiveness-ratio when vertebral fractures were incorporated. To allow comprehensive statements on the cost-effectiveness of FPE more homogenous studies with statements on specific fracture types are needed.

## Supplementary Information

**Additional file 1.**

## Data Availability

Datasets used and analysed during the current study are included in this published article and its supplementary files. The datasets used for the probabilistic sensitivity analysis are available from the corresponding author on reasonable request.

## Abbreviations

CI: Confidence interval
DRG: Diagnosis-related groups
e.g.: For example
FaME: Falls Management Exercise Program
FPE: Fall prevention exercise
ICD: International classification of diseases
ICER: Incremental cost-effectiveness-ratio
M: Men
Mio: Million
Non-inst.: Non-institutionalized
OEP: Otago Exercise Program
QALY: Quality-adjusted life year
SHI: Statutory health insurance
W: Women
WTP: Willingness to pay
