# Supplementary material for: Cost-effectiveness of group-based exercise to prevent falls in elderly community-dwelling people
Source: BMC Geriatr. 2021 Jul 26;21:440. doi: 10.1186/s12877-021-02329-0 (PMC8314607; doi:10.1186/s12877-021-02329-0)
Supplement: Supplementary file 1 — Additional file 1. [file 12877_2021_2329_MOESM1_ESM.docx]

Appendix

Table A1: Hospital costs of hip fracture treatment

| **Procedure** | **Costs** (€/Procedure) | **%** | **Partial costs** (€/hip fracture) | **Reference** |
| --- | --- | --- | --- | --- |
| Dynamic hip screw/angle plate | 6,855.97 | 0,05 | 342.80 | [42-44] |
| Intramedullary stabilization | 6,855.97 | 0,443 | 3.037.19 |  |
| Screw fixation^1^ | 5,907.69* | 0,047 | 277.66 |  |
| Total hip endoprothesis | 8,029.36 | 0,115 | 923.38 |  |
| Femoral head prosthesis | 6,731.90 | 0,026 | 175.03 |  |
| Dual head prosthesis | 8,029.36 | 0,324 | 2,601.51 |  |
| Total |  |  | 7,357.57 |  |
| Out of pocket payments |  |  | 111.04 |  |
| Total |  |  | **7,246.53** |  |

^1^Average value for relevant screw procedures

Table A2. Outpatient costs for hip fracture treatment

| **Procedure** | **Costs** (€/Procedure) | **Amount** | **Costs** (€/hip fracture) | **Reference** |
| --- | --- | --- | --- | --- |
| Physiotherapy | 19.33 | 30 | 579.90 | [50, 53] |
| Home visit | 11.27 | 30 | 338.10 | [50, 53] |
| Massage | 13.18 | 10 | 131.80 | [50, 53] |
| *Physiotherapy total* |  |  | *1,049.80* |  |
| Insurance lump sum per patient | 22.84 | 2 | 45.68 | [52] |
| Insurance lump sum per consultation | 1.95 | 4 | 7.80 | [52] |
| Home visit | 22.94 | 4 | 91.76 | [50, 52] |
| Blood test | 1.10 | 1 | 1.10 | [50, 52] |
| Tramadol (50ml) | 18.04 | 1 | 18.04 | [50, 51] |
| Phenprocoumon (50 tablets) | 9.54 | 1 | 9.54 | [51] |
| X-ray follow-up | 8.44 | 1 | 8.44 | [52] |
| Consiliary x-ray assessment | 11.90 | 1 | 11.90 | [52] |
| *Physician care total* |  |  | *199.26* |  |
| Total |  |  | ***1,249.06*** |  |
| Total (after consideration of out of pocket payments)^1^ |  |  | ***1,114.08*** |  |

^1^corresponds to the cost of the SHI

Table A3. Costs of outpatient long-term care prior hip fracture

| **Level of care** | **Costs for family care** (€) | **Costs for nursing care** (€) | **Proportion family care** | **Proportion nursing care** | **Partial costs** (€) | **Reference** |
| --- | --- | --- | --- | --- | --- | --- |
| I^1^ | - | - | - | - | - | [28, 42, 54] |
| II | 316 | 689 | 0.38 | 0.14 | 218.22 |  |
| III | 545 | 1,298 | 0.20 | 0.11 | 245.53 |  |
| IV | 728 | 1,612 | 0.08 | 0.06 | 151.54 |  |
| V | 901 | 1,995 | 0.02 | 0.02 | 64.27 |  |
| Total (1 month) |  |  |  |  | **679,56** |  |
| Total (6 months)^2^ |  |  |  |  | **4,077.33** |  |

^1^Level of care I was not incorporated since there were no data available

^2^Long-term care costs were incorporated in our model partially according to the age-specific care rate in the cohort

Table A4. Costs of outpatient long-term care post hip fracture

| **Level of care** | **Costs for family care** | **Costs for nursing care** | **Proportion family care^2,3^** | **Proportion Nursing care^2,3^** | **Partial costs** (€) | **Reference** |
| --- | --- | --- | --- | --- | --- | --- |
| I^1^ | - | - | - | - | - | [28, 42, 54] |
| II | 316 | 689 | 0.19 | 0.06 | 101.41 |  |
| III | 545 | 1,298 | 0.19 | 0.11 | 242.02 |  |
| IV | 728 | 1,612 | 0.17 | 0.12 | 312.16 |  |
| V | 901 | 1,995 | 0.10 | 0.08 | 235.80 |  |
| Total (1 month) |  |  |  |  | **891.39** |  |
| Total (6 months)^b^ |  |  |  |  | **5,348.34** |  |

^1^Level of care I was not incorporated since there were no data available

^2^Long-term care costs were incorporated proportionately according to the age-specific care rate in the cohort

^3^Proportions post hip fracture were derived from Muller-Mai et al and adjusted to the new implemented care level system in Germany in 2017 [42].

Table A5. Costs of inpatient long-term care

| **Level of care** | **Costs** (€/month)^1^ | **Proportion** | **Partial costs** (€/month) | **References** |
| --- | --- | --- | --- | --- |
| I | 125 | 0.01 | 1.15 | [28, 54] |
| II | 770 | 0.21 | 164.95 |  |
| III | 1,262 | 0.32 | 400.23 |  |
| IV | 1,775 | 0.30 | 525.50 |  |
| V | 2,005 | 0.16 | 327.57 |  |
| Total (1 month) |  |  | **1,419.39** |  |
| Total (6 months) |  |  | **8,516.36** |  |

^1^Monthly claims of the insured to the SHI [34]

Table A6. Long-term care rates prior and post hip fracture

| **Age**  (years) | **Long-term care rate** (w/m, non-inst.) | **Long-term care rate** (w/m, not-inst.) | **References** |
| --- | --- | --- | --- |
|  | Prior hip fracture | Post hip fracture |  |
| 75-79 | 0,1/0,09 | 0,19/0,17 | [28, 36] |
| 80-84 | 0,2/0,16 | 0,42/0,37 |  |
| 85-89 | 0,39/0,3 | 0,61/0,51 |  |
| 90+ | 0,63/0,4 | 0,84/0,7 |  |

Abbreviations. Non-inst.: non-institutionalized

Figure A1. Transition state Markov model for the incorporation of other possible transitions to nursing home (structural sensitivity analysis I) [24]

Abbreviations. Non-inst.: non-institutionalized

Figure A2. Transition state Markov model for the additional incorporation of vertebral fractures (structural sensitivity analysis II) [24]

Abbreviations. Non-inst.: non-institutionalized

Table A7: Additional Input data on nursing home admissions unrelated to hip fracture (Structural Sensitivity analysis I)

| **Age**  (years) | **Admissions to nursing home**  (%, non-inst., w/m) [56] | **Admissions to nursing home**  (%, non-inst., w/m) [56] |
| --- | --- | --- |
|  | Prior hip fracture | Post hip fracture^1^ |
| 75-79 | 0.002/0.002 | 0.002/0.002 |
| 80-84 | 0.016/0.01 | 0.014/0.011 |
| 85-89 | 0.013/0.011 | 0.013/0.010 |
| 90-94 | 0.042/0.035 | 0.040/0.034 |
| 95+ | 0.041/0.034 | 0.039/0.031 |

^1^Adjusted for higher hip fracture mortality post hip fracture (see table 1)

Abbreviations. w: woman; m: men; non-inst.: non-institutionalized

| **Age** (years) | **Vertebral fracture** (%, non-inst., w/m)  [57] | **Re-fracture vertebral/hip*** (%, non-inst., w/m)  [57] | **Re-fracture vertebral/hip*** (%, nursing home, w/m)  [57] | **Nursing home admission** (%, w/m)  [57] |
| --- | --- | --- | --- | --- |
|  |  | Months 1-6 post fracture | Months 1-6 post fracture | Months 1-6 post vertebral fracture |
| 75-79 | 0.001/0.002 | 0.016/0.013 | 0.063/0.029 | 0.048/0.041 |
| 80-84 | 0.001/0.003 | 0.031/0.020 | 0.076/0.041 | 0.078/0.076 |
| 85-89 | 0.002/0.003 | 0.05/0.028 | 0.081/0.036 | 0.121/0.073 |
| 90-94 | 0.002/0.003 | 0.07/0.037 | 0.087/0.042 | 0.150/0.129 |
| 95+ | 0.003/0.003 | 0.088/0.049 | 0.066/0.013 | 0.187/0.092 |
|  |  | Months 7+ post fracture | Months 7+ post fracture | Months 1-6 post hip/vertebral fracture |
| 75-79 |  | 0.004/0.002 | 0.018/0.012 | 0.067/0.066 |
| 80-84 |  | 0.007/0.004 | 0.022/0.017 | 0.117/0.096 |
| 85-89 |  | 0.012/0.006 | 0.025/0.02 | 0.147/0.106 |
| 90-94 |  | 0.018/0.001 | 0.027/0.023 | 0.18/0.117 |
| 95+ |  | 0.022/0.014 | 0.023/0.021 | 0.201/0.186 |
|  | **Vertebral fracture mortality** (%, non-inst., w/m)^1^ [21, 57] | **Costs of treatment**  [49, 58] | (€) |  |
| 75-79 | 0.031/0.091 | Vertebral fracture | 6,515 |  |
| 80-84 | 0.051/0.131 | Hip/Vertebral fracture | 10,123 |  |
| 85-89 | 0.079/0.129 |  |  |  |
| 90+ | 0.150/0.133 |  |  |  |

Table A8: Additional input data on vertebral fractures (Structural Sensitivity analysis II)

Re-fracture probabilities are based on the hip fracture probabilities of the base-case analysis and weighted together with the vertebral fracture probabilities. For input data on hip fractures see table 1.

^1^Months 1-6 post vertebral fracture

Abbreviations. w: woman; m: men; non-inst.: non-institutionalized

Table A9. Results of the additional structural sensitivity analyses

|  | **ICER** (w, €/avoided hip fracture) | **ICER** (m, €/avoided hip fracture) |
| --- | --- | --- |
| Base Case | 52,864 | 169,805 |
| Additional Analysis I: Admissions to nursing homes unrelated to hip fractures | 59,873 | 172,248 |
| Additional Analysis II: Additional incorporation of vertebral fractures | 46,617^1^ | 78,936^1^ |

^1^for this analysis the outcome was reported as costs in € / avoided hip or vertebral fracture.

Abbreviations. w: woman; m: men;

Figure A3. Results of the deterministic sensitivity analysis (women)

Varied data is provided in table 1. The tornado diagram is showing the 7 parameters with the highest impact on results.

Abbreviations. ICER: Incremental cost-effectiveness-ratio

Figure A4. Results of the deterministic sensitivity analysis (men)

Varied data is provided in table 1. The tornado diagram is showing the 7 parameters with the highest impact on results.

Abbreviations. ICER: Incremental cost-effectiveness-ratio
